# Chromatin States Accurately Classify Cell Differentiation Stages

**DOI:** 10.1371/journal.pone.0031414

**Published:** 2012-02-20

**Authors:** Jessica L. Larson, Guo-Cheng Yuan

**Affiliations:** 1 Department of Biostatistics, Harvard School of Public Health, Boston, Massachusetts, United States of America; 2 Department of Biostatistics and Computational Biology, Dana-Farber Cancer Institute, Boston, Massachusetts, United States of America; Newcastle University, United Kingdom

## Abstract

Gene expression is controlled by the concerted interactions between transcription factors and chromatin regulators. While recent studies have identified global chromatin state changes across cell-types, it remains unclear to what extent these changes are co-regulated during cell-differentiation. Here we present a comprehensive computational analysis by assembling a large dataset containing genome-wide occupancy information of 5 histone modifications in 27 human cell lines (including 24 normal and 3 cancer cell lines) obtained from the public domain, followed by independent analysis at three different representations. We classified the differentiation stage of a cell-type based on its genome-wide pattern of chromatin states, and found that our method was able to identify normal cell lines with nearly 100% accuracy. We then applied our model to classify the cancer cell lines and found that each can be unequivocally classified as differentiated cells. The differences can be in part explained by the differential activities of three regulatory modules associated with embryonic stem cells. We also found that the “hotspot” genes, whose chromatin states change dynamically in accordance to the differentiation stage, are not randomly distributed across the genome but tend to be embedded in multi-gene chromatin domains, and that specialized gene clusters tend to be embedded in stably occupied domains.

## Introduction

Multi-cellular organisms are composed of diverse cell types that, despite sharing the same genome, are programmed with distinct gene expression patterns. How such diversity is regulated mechanistically is a fundamental biological question. Eukaryotic DNA is packaged in chromatin. The fundamental unit of chromatin is nucleosome, a histone octamer, which wraps around 147 bp DNA. The N-terminal tails of histone proteins are decorated by different marks resulting from covalent modifications. The combinatorial patterns of these marks, which we refer to as the chromatin states, may recruit specific regulatory proteins, which in turn control transcription [1], [2].

Recent genome-wide location studies have identified distinct chromatin states that demarcate regulatory elements [3], [4], [5], [6], [7]. Furthermore, the chromatin states changes significantly between different cell types, in accordance with gene expression level changes [3], [8], [9], [10], [11], [12], [13], [14], [15], providing strong evidence that the chromatin states play an important role in development. On the other hand, these studies have been limited to comparing a small number of cell types. As a result, it is difficult to evaluate to what extent cell lineages are associated with chromatin states. Characterization of the molecular signatures associated with cell lineages will not only provide insights into the transcription control but help identifying the cell-of-origin, which is an important task for many diseases. For example, an intensively investigated area of cancer research is whether a tumor is originated from cancer stem cells or normal differentiated cells. Understanding the origin of cancer cells has important implications in developing therapeutic methods.

The idea of using genomic data to classify cell lineages is not new. There have been extensive studies based on gene expression data (reviewed by [16]). However, one major limitation is that gene expression levels do not inform us the underlying controlling mechanism, which is fundamental for understanding developmental processes and diseases. For example, gene expression analyses have discovered the intriguing phenomenon that tumors with poor clinical outcome often display a signature that is similar to stem cells [17]. However, the underlying mechanism remains incompletely understood. Recently, it has been shown that the similarity is mainly due to the activity of the MYC regulatory module rather than the core module targeted by pluripotent factors [18].

**Figure 1:**
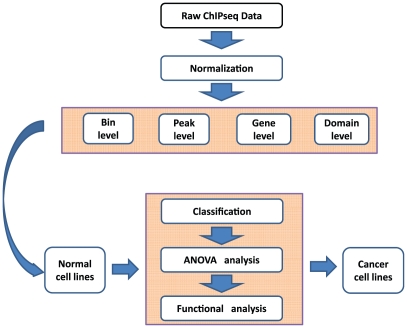
Overview of the data analysis strategy. ChIPseq data of 5 histone modifications in 27 human cell lines were obtained from the public domains and analyzed independently using four different representations (bin, gene score, and chromatin state level). For each representation, a support vector machine (SVM) model was used to classify cell differentiation status from histone modification data. “Hotspot” genes or bins were detected by ANOVA and further investigated by functional genomic tools. The SVM model obtained from normal cell lines was used to classify the differentiation status of cancer cell lines.

Recently, a large amount of genome-wide histone modification data have been generated and made publicly available, in part due to the effort of ENCODE and Roadmap Epigenomics consortia [15], [19]. These data have provided a great opportunity to identify general principles of chromatin regulation. In this paper, we will focus on evaluating the association between chromatin states and cell differentiation stages. To this end, we assembled a large dataset from the public domain of genome-wide locations of 5 histone modifications in 27 human cell lines and analyzed the data independently using four different spatial representations (see Figure 1 for a schematic overview). We found that cell differentiation status can be classified with nearly 100% accuracy from chromatin states alone, that chromatin state switches are frequently associated with multi-gene domains, and that the cancer cell lines have similar chromatin states as differentiated cells.

## Results

### An assembly of genome-wide data for 5 histone modifications in 27 human cell lines

We collected genome-wide histone modification data from NIH Epigenome Roadmap [20] and other public domains [4], [11], [13], [21]. We focused on five histone modifications that have been profiled extensively, including four associated with active genes (H3K4me1, H3K4me3, H3K36me3, H3K9ac) and one associated with gene silencing (H3K27me3). We focused on 27 human cell lines for which data for all five modifications are available, including 24 normal and 3 cancer cell lines (Table S1). Of the 24 normal cell lines, five are pluripotent cells (P), four are multipotent (M), which may further differentiate into multiple cell-types; and the others are either unipotent progenitors or terminally differentiated cells, which were grouped together (U/D). The three cancer cell lines are: K562 (chronic myelogenous leukemia), HeLa (cervical cancer), and VCaP (prostate cancer).

To systematically compare different length scales, we analyzed the data independently based on three different representations, corresponding to increasing complex signatures: (1) the bin level sequence reads; (2) the gene-level summary scores associated with each histone mark; and (3) the combinatorial patterns of multiple histone marks referred to as the chromatin states (see Materials and Methods for more details).

In previous work [22], we developed a hidden Markov model (HMM) to identify chromatin states, treating each gene as a unit. Here we applied this approach to analyze the ChIP-seq dataset for the 27 human cell lines. As before, we determined the number of chromatin states based on the gap statistic [23] (see Materials and Methods for details), and found that the optimal number of clusters is 3 (Figure S1). This is the same number of chromatin states we identified previously for mouse data [22]. We found the genome-wide pattern is described by three HMM states: active (associated with active marks), non-active (associated with H3K27me3), and null (lack of both active and repressive marks) (Table S2). We applied a common model to infer genome-wide chromatin states in all cell lines (Table S3). As the non-active state is associated with relative high density of both H3K27me3 and H3K4me3, we were also interested to test if there was a significant overlap with the bivalent domains. Indeed, we found that 25.8% of genes containing bivalent domains correspond to the nonactive state (Figure S2). On the other hand, we also found that 67.0% of bivalent domains correspond to the active state. These genes are typically associated with higher density of additional active marks such as H3K4me1 and H3K36me3. This observation is consistent with a recent study showing that a subset of genes marked by bivalent domains are actively transcribed [24], but it also suggests that the chromatin states may be further refined.

### Histone modification patterns accurately classify cell differentiation status

We wanted to compare our three methods of analyzing ChIPseq data (i.e., the bin, gene, and chromatin state levels) to determine which one is the ‘best’ at classification of cell lineages, which were grouped by the differentiation status: P, M, or U/D. For each representation, we built a support vector machine (SVM) model to classify the membership of a cell line based on the histone modification data (see Materials and Methods). In order to avoid overfitting, we evaluated the classification accuracy using leave-one-out cross-validation. The classification accuracy was quantified by the percentage of agreement between the model predicted and true differentiation status. Surprisingly, we found that all representations led to 100% accuracy (compared to 62% obtained by using the null model, which classifies every cell line as the largest group, i.e., U/D). To see if that result is robust or depends on a particular approach, we repeated the analysis by using the radial kernel function instead and again achieved 100% classification accuracy. These results strongly suggest that distinct chromatin states are associated with different differentiation statuses. This striking difference prompted us to further dissect biological features of these distinctive chromatin states and to pursue the utility of chromatin states for the classification of poorly characterized cell-types.

### Numerous locations of epigenetic difference found between cell lines

To gain functional insights, we searched for regions that are most discriminative across different cell lineages: P, M, or U/D. To this end, we applied a permutation ANOVA F- test and selected those regions (bins or genes) that are differentially modified (FDR<0.05) (See Materials and Methods for detail). Indeed we found extensive differences at each level.

**Bin level:** Our ANOVA analysis identified 249,705 differential bins for the H3K4me1 analysis, 21,224 bins for H3K4me3, 5,354 bins for H3K9ac, 25,385 bins for H3K27me3, and 69,373 bins for H3K36me3 (Table S4). On the other hand, only 7 differential bins were common to all five modifications (Figure S3a), consistent with the previous results that they each demarcate different regions [4], [9]. While the H3K27me3 and H3K4me1 bins are distributed quite uniformly across the genome, the other three modifications showed a strong bias toward coding regions, promoters, CpG islands and shores (Figure S3b). Interestingly, while the mean occupancy levels of H3K4me3 and H3K9ac are highest in promoter regions, the variance can be high in coding regions as well. Conversely, while H3K36me3 is known to be mainly targeted toward coding regions, the variance can also be high in promoter regions.

**Figure 2:**
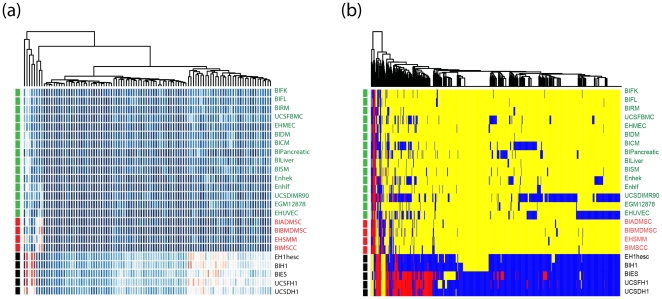
Differentiation related variation of histone modification patterns. (a) Heatmap showing the gene-level H3K4me1 scores for the 100 most significantly different genes. (b) Heatmap of the chromatin states for the 722 “hotspot” genes, whose chromatin states are significantly different across differentiation statuses. Red – active state; yellow – null state; blue – nonactive state. The cell line information is shown at both sides of the heatmap and color-coded by the differentiation status (black – pluripotent cells (P); red – multipotent (M); green – unipotent/differentiated (U/D)).

**Gene level:** We found 2,501 genes that are differential based on their H3K4me1 gene-level score, 2,119 genes for H3K4me3, 368 genes for H3K9ac, 569 genes for H3K27me3, and 4,731 genes for H3K36me3 (Table S5). For most of these genes, the gene-level scores are higher in pluripotent cells than multipotent and differentiated cells (Figure 2a, and Figures S4b–e). Again, the overlap between different modifications is low: only 4 genes were common to all modifications (Figure S4a).

**Chromatin state level:** By applying our ANOVA analysis to the chromatin state information obtained by our hidden Markov model, we found 722 differential genes, which were analyzed further. We call these genes the “hotspot” genes to highlight their role in chromatin state remodeling (Table S6). We observed two main patterns of chromatin state switch during cell differentiation (Figure 2b): 1) most “hotspot” genes are in the nonactive state in pluripotent cells and switch to the null state in differentiated cells; 2) a smaller subset of genes are in the active state in pluripotent cells and switch to another state during differentiation. Interestingly, most “hotspot” genes are in the null state in U/D cells. A closer examination suggested that a number of additional genes were also marked by H3K4me3 and H3K9ac, although the gene-level scores tended to be lower compared to the active state. 244 of these hotspot genes display distinct chromatin state pattern in ES cells compared with the other cell lines (Table S7). Among these 244 genes, 209 are in the non-active state in ES cells.

**Figure 3:**
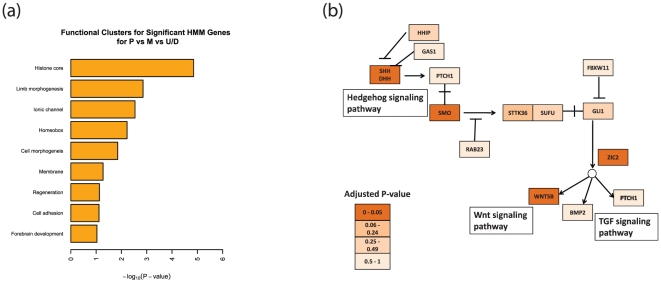
Functional enrichment analysis of the “hotspot” genes. A total of 722 “hotspot” genes were identified by applying ANOVA analysis to the chromatin states. Enriched functions and pathways were identified by using DAVID. (a) Representative enriched functional categories. (b) The Hedgehog signaling pathway is significantly enriched (p-value = 0.008). The genes were color-coded based their corresponding adjusted p-values obtained from ANOVA analysis.

To gain functional insights, we applied the Database for Annotation, Visualization and Integrated Discovery (DAVID) [25] to identify enriched functional categories that are associated with the “hotspot” genes. We found significant enrichment of genes associated with the Homeobox, cell-cell signaling, or neuron development (Figure 3a), consistent with an important role of chromatin state remodeling during development.

In addition, those “hotspot” genes that undergo different remodeling paths during cell differentiation tend to be associated with different biological functions. Specifically, the genes that are active in pluripotent cells (such as HIST1H4F) tend to be associated with chromatin organization and methylation, the genes that are non-active in pluripotent cells (such as the Hox genes) are usually involved in organism development, while the genes that are in the null state in pluripotent cells (such as APOL6) seem to participate in diverse biological processes (Figure S5).

Our functional analysis also identified four signaling pathways enriched in the “hotspot” genes: neuroactive ligand-receptor interaction (p-value = 0.0017), calcium-signaling (p-value = 0.006), hedgehog-signaling (p-value = 0.008), and TGF-Beta signaling pathway (p-value = 0.013). The hedgehog-signaling pathway is particularly interesting since it plays an essential role in embryo segmentation and is conserved from flies to humans [26]. There are four “hotspot” genes (adjusted p-value<0.05) in this pathway, including SHH, SMO, WNT, and ZIC2 (Figure 3b), suggesting that chromatin state remodeling may play an important role in regulating the cell-type specific activity of this important pathway. Likewise the TGF-Beta signaling pathway is involved in embryo differentiation, left-right axis determination, apoptosis and mesoderm/endoderm development [27].

### Coordinated switches in chromatin domains

We were interested in finding the extent to which developmentally related chromatin state remodeling was spatially coordinated. As before [22], we merged neighboring genes of the same state into blocks, and identified chromatin domains as those blocks that were significantly larger than expected by chance (see Materials and Methods for detail). We found that 1,874 genes were contained in a significant domain in at least two cell lines. In the following we refer to these as the domain-associated genes.

1,874 genes are found in the domain-associated group. Interestingly, we found there is a significant overlap between this group and the “hotspot” genes identified by our ANOVA analysis. 11.2% of the “hotspot” genes fall into this category (p-value = 0.0346). In addition, the genes that are significant only in one modification are also strongly associated (p-value<0.01). These results suggest that chromatin state remodeling does not occur by random but is regulated in a spatially coordinated manner, perhaps through active maintenance of the domain boundaries.

**Figure 4:**
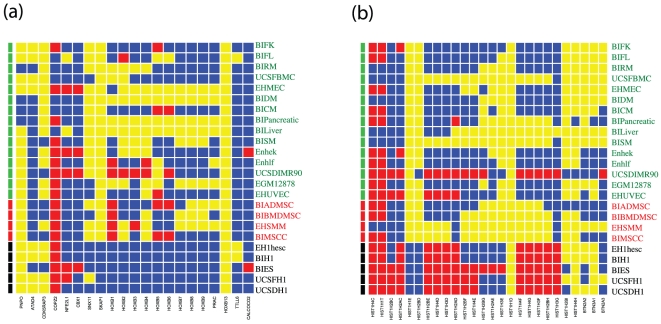
Representative chromatin domains identified by the hidden Markov model. Heatmap of the chromatin state distribution at the local genomic loci. (a) the HOXB gene cluster; (b) The histone gene cluster. Genes are ordered according to their genomic positions. Red – active state; yellow – null state; blue – nonactive state. The cell line information is shown at both sides of the heatmap and color-coded by the differentiation status (black – plutipotent (P) cells; red – multipotent (M); green – unipotent/differentiated (U/D)).

One of the classical examples of chromatin domains is the HOXB gene cluster (Figure 4a). In particular, in the ES cell lines, the genes found in this domain are in the non-active state, which is characterized by high H3K27me3 occupancy. In differentiated cells, the HOXD genes switched to the null state. The histone gene cluster on chromosome 6 also undergoes a domain-level change during cellular differentiation (Figure 4b). These genes are mostly in the active state in ES cells, and then some of them (HIST1H14D to HIST1H3G) switch to a null state for most of the multipotent cell lines. There is then a switch back to a mostly non-active state for the unipotent/differentiated cell lines.

A small subset (containing 171 genes) of the domain-associated genes are persistent in the sense that they are embedded in a domain in almost every cell type (n>21). This set of genes consists of specialized gene clusters, such as the olfactory receptor (OR) clusters, the keratin-associated protein cluster, and the Leukocyte Ig-like receptors (LIRs) cluster. These gene clusters tend to be silenced in almost every cell type except for few highly specialized cell types such as the olfactory receptors, keratin cells, or leukocytes. Consistent with gene silencing, these genes are not associated with active histone modifications.

### Histone modification patterns in cancer cells are similar to differentiated cell types

The high accuracy of our chromatin-based classification models suggests that it may be useful for classification of poorly characterized cell-types. Cancer cells do not follow normal cell differentiation pathway and their lineages are poorly characterized. It has been noted that cancer cells often display characteristics similar to stem cells. Our above results suggested that chromatin states can be used to provide mechanistic insights into the relationship between cancer and stem cells. To this end, we applied our classification models to three cancer lines (K562, HeLa, VCaP), for which we were able to obtain the histone modification data. Strikingly, each cell line was unequivocally classified as U/D (Table 1), suggesting significant and robust chromatin structural differences between cancer and stem cells (either pluripotent or multipotent cells).

**Table 1:** Outcome of classifying the differentiation status of three cancer cell lines (K562, HeLa, and VCaP) by applying the support vector machine to histone modification data at different levels.

	K562	HeLa	VCaP
**Bin (5 models)**	0/0/5	1/0/4	0/0/5
**Gene, single mark (5 models)**	0/0/5	0/0/5	0/0/5
**Chromatin state (1 model)**	0/0/1	0/0/1	0/0/1

The results are represented as three numbers, corresponding to the number of models for which the cell line classified as pluripotent (P), multipotent (M), or unipotent/differentiated (U/D), respectively.

**Figure 5:**
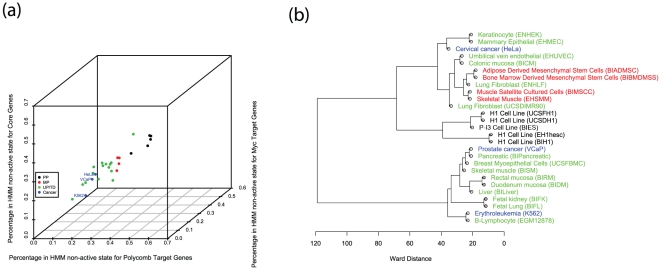
Cancer cells display similar chromatin state patterns as fully differentiated cells. (a) A scatter plot of cell-type specific chromatin states associated with the three ES regulatory modules. The chromatin state of a module is summarized by the fraction of non-active-state genes. Each data point corresponds to one cell-type and is color-coded according to the differentiation status. The three cancer cells are labeled. (b) Hierarchical clustering of the 27 cell lines based on the chromatin states.

We further investigated the association between chromatin state changes and known regulatory modules. In a recent study, Kim et al. identified three ES regulatory modules based on protein-DNA interaction data [18]. These modules correspond to target genes of core ES cell regulators such as OCT4 and NANOG (called the Core module); of Polycomb group complexes (called the PRC module); and of the MYC related regulators (called the MYC module), respectively. These authors found that the gene expression patterns of cancer and stem cells are similar for the MYC module but significantly different for the other two modules. These observations led us to compare the chromatin state organization at these modules between different cell-types. As a simple quantitative metric, we evaluated the fraction of genes within each module falling into the non-active state (Figure 5a). The difference between different cell-lineage groups is apparent. Furthermore, the cancer cell lines seem to be distinct from the stem cells (either pluripotent or multipotent).

To test whether the activity of these modules is sufficient to explain the differences among differentiation stages, we built a multinomial logistic regression model to classify differentiation status solely based on the three module-specific chromatin signatures (see Materials and Methods). This simple model already has 88% classification accuracy. We then applied this module-based model to classify the differentiation status of the three cancer cell lines. The results are similar to those obtained from the full SVM models. All three cancer cell lines (HeLa and VCaP) were classified as U/D. Therefore, the differences between cancer cells and stem cells can be interpreted simply by the differential activity of the three ES regulatory modules.

We divided the cell lines into groups of similar chromatin states by hierarchical clustering (Figure 5b). Both pluripotent cells and multipotent cells form distinct clusters, providing additional support to our classification results. Interestingly, two of the three U/D cell lines that were clustered together with multipotent cells may also be viewed as somewhat undifferentiated, since they are both fibroblasts and can undergo further differentiation and become more specialized. On the other hand, the three cancer cell lines are not only clustered with U/D cell lines but also positioned next to the cell lines from the same germ-layer.

In addition, we found that 290 genes have different chromatin state patterns compared to the normal differentiated cells (Table S8). 197 of these genes are in the non-active state in cancer cells. Only 10 genes from this list overlap with the “hotspot” genes that differentiating normal cell lines across differentiation stages, suggesting that these cancer cell lines contain additional chromatin signature that is distinct from normal differentiated cell lines. Functional analysis suggested that these genes are enriched with genes associated with nucleosome organization (p-value = 0.0002).

### Intrinsic chromatin state variability is associated with multiple human diseases

Aberrant epigenetic regulation has been linked to many diseases, including cancer, endocrine, and respiratory diseases [28], [29]. We hypothesized that such alteration may be partially contributed to intrinsic variability that occurs during normal differentiation and reasoned that, if so, the “hotspot” genes identified by our study should be significantly associated with various diseases.

By using the DAVID analysis tool again, we found that the “hotspot” genes are significantly associated with chemical dependency diseases (adjusted p-value = 0.0075; associated genes include HTR2C, CHRNA4, and APOE), developmental disease (adjusted p-value = 0.0081; associated genes include NLGN3, and GLO1), and physiological diseases (adjusted p-value = 0.0092; associated genes include APOL2 and OXT). In contrast, we did not find any cancer type with significant association, with the lowest adjusted p-value at 0.92 (for prostate carcinoma). This lack of association further supports our view that chromatin states in cancer cells are fundamentally different from stem cells.

## Discussion

Through a systematic analysis of a large dataset containing 5 histone modifications in 27 human cell lines at three different representations, we found that the chromatin states can classify cell differentiation stages with nearly 100% accuracy. To our knowledge, this is the first study to classify cell differentiation stages based on chromatin information. Our results strongly suggest that the chromatin states are co-regulated at each developmental stage. We identified 722 “hotspot” genes, whose chromatin states are significantly associated with the differentiation stages. These genes are enriched with functions related to development, cell-cell signaling, and chromatin structure.

The success of our classification model led us to test if it can be used to gain insights into the origin of cancer cells. To this end, we applied our model to classify three cancer cell lines for which we obtained genome-wide histone modification data from the public domains, including K562 (chronic myelogenous leukemia), HeLa (cervical cancer), and VCaP (prostate cancer). We found that these cancer cells can be unequivocally classified as differentiated cells based on the chromatin states, and that the differences between cancer and stem cells can be interpreted simply by using the three regulatory modules identified in ES cells [18]. Furthermore, all three cancer cell lines were clustered next to cell lines from the same germ layer, suggesting they may indeed originate from normal differentiated cells. Our analysis has provided new insights into the different regulatory mechanisms between cancer and stem cells. In future work, it will be very interesting to characterize the chromatin states in tumor samples and to investigate to what extent the chromatin states are associated with clinical outcome.

Recent studies have identified large-scale domains formed by various epigenetic marks [12], [13], [30]. A major difference between our and the aforementioned studies is that we treat each gene as a distinct unit, thereby ignoring the interruption of histone modification patterns at intergenic regions which may not be relevant for gene regulation. This allowed us to identify active domains despite the absence of active histone marks in intergenic regions. We also found that the “repressive” domains can be further divided into two types: “non-active” and “null”. Their main difference is that, while the “non-active” domains are associated with high H3K27me3 activity, the null domains do not appear to be associated with any histone mark. It will be interesting to further investigate that the null domains may be associated with certain repressive histone marks that are not included here. These two domain types also differ functionally. The non-active domains are associated with poised gene activation, and the null domains seem to be able to achieve more stable gene silencing and therefore are desirable for the regulation of highly specialized gene clusters such as keratin and olfactory receptors.

The extensive presence of null domains was first discovered in mouse ES cells, where we found that many OR genes were associated with this pattern [22]. The functional relevance has been supported by a recent experimental study, which showed that the transition from the null state to a new state marked by H3K9me3 and H4K20me3 plays an important role in the development of olfactory neurons [31]. Our analysis here has extended these previous studies, suggesting that transition from null states may be a general mechanism for control of cell-type specific gene regulation. It will be very interesting to experimentally test this hypothesis in future studies.

## Materials and Methods

### Raw data processing

**Bin level:** Raw ChIP-seq data were divided into 100 bp bins via BEDTools [32] and normalized to reads per million reads (RPM) to allow comparison across cell lines. Bins that overlapped 50% or more with known repetitive regions [33] were removed from further analysis. Remaining bins were merged into 1 kilobase (Kb) regions. The bins with no reads in any cell line were removed. Ultimately, we were left with 2,388,489 bins for further analysis.

**Gene level:** Gene annotations were based on Refseq [34]. Promoter regions were defined as the [−2 Kb, +2 Kb] region with respect to transcription start sites (TSS). For H3K4me1, H3K4me3, H3K27me3 and H3K9ac, the gene-level scores were defined by averaging normalized sequence reads over each gene promoter. For H3K36me3, it has been shown that the sequence reads are highest at 80–95% of the coding region of a gene [4]; the gene-level scores were defined as by averaging over these regions. After removing the genes that substantially overlap with repetitive regions, we were left with 18,385 genes for further investigation.

**Chromatin state level:** The chromatin states were detected using a hidden Markov model (HMM) as previously described [22]. Briefly, the HMM combines gene-level scores for all five histone modifications (the emission variable) and classifies them as distinct chromatin states (the hidden variable). For simplicity, the emission probability is modeled by a multivariate Gaussian distribution with no covariance structure. To determine the optimal number of chromatin states, we clustered these five-dimensional vectors using the k-means average agglomeration clustering method. The optimal cluster number k was selected using the gap statistic [23] defined as
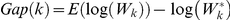
where W^*^_k_ is the observed within-cluster sum of squares around the clusters means for one run, and E(⋅) represents the mean value for 1000 random bootstrap permutations. The gap statistic is maximized at k = 3 (Figure S1).

We initially fit a three-state model separately for each cell line on chromosome 22, and used the expectation-maximization (EM) algorithm to estimate these model parameters. These cell-type specific models were averaged to obtain a single common model, which was then applied to determine the genome-wide chromatin states in the 27 cell lines via the Viterbi algorithm [35].

The bivalent genes were identified similar to the traditional definition [3], but with the modification necessary to map to the bin-level data. Specifically, we identified all the bivalent bins, that is, those 1 Kb bins that overlap with both H3K4me3 and H3K27me3 peak locations, where the peak locations were detected by adapting the CisGenome algorithm to bin-level data [36]. The bivalent genes were identified as those whose promoter overlaps with at least one bivalent bin.

**Domain level:** For each cell line, consecutive genes sharing the same chromatin states were merged as domains. As in previous work [22], we further used a likelihood-ratio test to identify significant domains in order to remove those domains that simply occur by chance. Specifically, for each domain, we calculated the ratio of the likelihood of observing its corresponding gene-level scores under the assumption that they were in the common chromatin state to the likelihood of observing the same data under the null hypothesis of no domain states. We estimated the null distribution based on 1,000 random permutations of all genes, and selected a cutoff domain size corresponding to the false discovery rate (FDR) at 0.05. Only those domains larger than the cutoff size were deemed significant and retained for further analysis. This analysis was repeated separately for each cell line; therefore the results are independent of the composition of cell lines in our assembly. A gene that is embedded in a significant domain in at least two cell lines is called domain-associated.

### Cell differentiation stage classification

We classified the differentiation status [pluripotent (P), multipotent (M), or unipotent/differentiated (U/D)] from histone modification data by using support vector machines (SVM) [37] using either linear
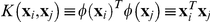
or radial
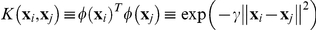
(where γ>0, and is estimated by cross-validation) kernel functions [38]. To determine which of the three ChIP-seq data representations (i.e., the bin, gene, and chromatin state levels) is most informative, we analyzed each representation independently. For the gene level analyses, all genes were used. For fair comparison, an equal number (i.e. equal to the number of genes) of most variable bins were used to construct the model. Calculations were done with the R package e1071 [39].

### A simple classification model based on three regulatory modules

Using the three ES regulatory modules (described in the main text), we fit the following multinomial logistic regression model on the 24 normal cell lines:
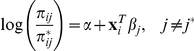
where π_ij_ is the probability that the i^th^ cell line (i = 1, 2, …, 24) is a member of differentiation class j (j = 1, 2, 3)and **x**_i_ is the percentage of genes in one of the three modules assigned to the null state. The i^th^ cell line will be classified as belonging to the j^th^ class, if π_ij_>0.5. It is possible that all π_ij_ are less than 0.5; in such a case, the differentiation status will be classified as unknown.

### Identification of differential regions

ANOVA analysis (F-test) was used to detect differential regions (bin/gene), where the histone modification patterns change significantly in accordance to the differentiation status. The null hypothesis was there were no systematic differences across different cell-lineage groups. This test was conducted via the multtest package in **R** [40]. To correct for the multiple hypothesis testing bias, we calculated the FDR values by using 100,000 random permutations [41]. A cutoff value of FDR = 0.05 was used to select differential regions.

## Supporting Information

Figure S1 **Determining number of chromatin states by using the gap statistic.** The expected and observed log (W_K_) values are shown in (a) for various levels of K (the number of clusters), where W_K_ is the pooled within cluster sum of squares around the cluster means. The number of clusters versus Gap(K), the difference between the observed and expected values (mean value for 1000 random bootstrap permutations), is shown in (b). According to these results, three is the optional number of clusters for our data.
(EPS)

Figure S2 **Proportion of bivalent genes in different chromatin states identified by HMM.** Red – active state; yellow – null state; blue – nonactive state.
(EPS)

Figure S3 **Overall distribution of the differential bins identified by the ANOVA analysis.** (a) A Venn diagram showing the overlap among different histone modifications. (b) Enrichment of various functional elements in the differential bins, where the enrichment scores were computed by ratio between the frequency of differential bins falling into one functional element category and that expected by chance.
(EPS)

Figure S4 **Overall distribution of the differential genes identified by applying the ANOVA analysis to gene-level scores.** (a) A Venn diagram showing the overlap among different histone modifications. (b–e) Heatmap of gene-level scores for the 100 most differential genes: (b) H3K4me3. (c) H3K9ac. (d) H3K27me3. (e) H3K36me3. The cell line information is shown at both sides of the heatmap and color-coded by the differentiation status (black – pluripotent (P) cells; red – multipotent (M); green – unipotent/differentiated (U/D)).
(EPS)

Figure S5 **Functional analysis of “hotspot” genes.** “Hotspot” genes were identified by applying ANOVA analysis to the chromatin states inferred by the hidden Markov model. These genes were further divided into three categories based on their corresponding state in the ES cells: red – active; yellow – null; blue – nonactive.
(EPS)

Table S1 **Description of the cell lines used in this study.** Code used for cell-type group: P – pluripotent cell; M – multipotent cell; U/D – unipotent/differentiated cell; C – cancer cell.
(XLSX)

Table S2 **Mean (standard deviation) gene-level histone modification scores associated with each chromatin state identified by the hidden Markov model.** The active state is associated with active marks, the non-active state is associated with the repressive mark H3K27me3, and the null state is not associated with any mark examined here.
(XLSX)

Table S3 **Cell-type specific chromatin states inferred by the hidden Markov model.** (1 – null; 2 – nonactive; 3 – active).
(XLSX)

Table S4 **List of differential bins for each histone modification mark.**
(XLSX)

Table S5 **List of differential genes based on gene-level scores for each histone modification mark.**
(XLSX)

Table S6 **List of “hotspot” genes whose chromatin states are significantly associated with differentiation status.**
(XLSX)

Table S7 **List of genes whose chromatin states are significantly different between pluripotent cells and other normal cell types.**
(XLSX)

Table S8 **List of genes whose chromatin states are significantly different between the normal and cancer cell lines.**
(XLSX)
